# Equation-Free Analysis of Two-Component System Signalling Model Reveals the Emergence of Co-Existing Phenotypes in the Absence of Multistationarity

**DOI:** 10.1371/journal.pcbi.1002396

**Published:** 2012-06-28

**Authors:** Rebecca B. Hoyle, Daniele Avitabile, Andrzej M. Kierzek

**Affiliations:** 1 Department of Mathematics, University of Surrey, Guildford, United Kingdom; 2 School of Mathematical Sciences, University of Nottingham, Nottingham, United Kingdom; 3 Division of Microbial Sciences, University of Surrey, Guildford, United Kingdom; University of Wisconsin-Madison, United States of America

## Abstract

Phenotypic differences of genetically identical cells under the same environmental conditions have been attributed to the inherent stochasticity of biochemical processes. Various mechanisms have been suggested, including the existence of alternative steady states in regulatory networks that are reached by means of stochastic fluctuations, long transient excursions from a stable state to an unstable excited state, and the switching on and off of a reaction network according to the availability of a constituent chemical species. Here we analyse a detailed stochastic kinetic model of two-component system signalling in bacteria, and show that alternative phenotypes emerge in the absence of these features. We perform a bifurcation analysis of deterministic reaction rate equations derived from the model, and find that they cannot reproduce the whole range of qualitative responses to external signals demonstrated by direct stochastic simulations. In particular, the mixed mode, where stochastic switching and a graded response are seen simultaneously, is absent. However, probabilistic and equation-free analyses of the stochastic model that calculate stationary states for the mean of an ensemble of stochastic trajectories reveal that slow transcription of either response regulator or histidine kinase leads to the coexistence of an approximate basal solution and a graded response that combine to produce the mixed mode, thus establishing its essential stochastic nature. The same techniques also show that stochasticity results in the observation of an all-or-none bistable response over a much wider range of external signals than would be expected on deterministic grounds. Thus we demonstrate the application of numerical equation-free methods to a detailed biochemical reaction network model, and show that it can provide new insight into the role of stochasticity in the emergence of phenotypic diversity.

## Author Summary

It is a surprising fact that genetically identical bacteria, living in identical conditions, can develop in completely different ways: for example, one subpopulation might grow very fast and another very slowly. These different phenotypes are thought to be one reason why bacteria that cause disease can survive antibiotic treatment or become persistent. This diversity of behaviour is usually attributed to the existence of multiple stable phenotypic states, or to the coexistence of one stable state with another unstable excited state, or finally to the possibility of the whole biochemical system that controls the phenotype being switched on and off. In this paper we describe a different scenario that leads to phenotypic diversity in two-component system signalling, a very common mechanism that bacteria use to sense external signals and control their response to changes in their environment. We use probability theory and equation-free computational analysis to calculate the average number of molecules of each chemical species present in the two-component system and hence show that sporadic production of either of two key chemical components required for signalling can delay the response to the external signal in some bacterial cells and so lead to the emergence of two distinct cell populations.

## Introduction

Phenotypic heterogeneity in populations of genetically identical (isogenic) cells is one of the major discoveries resulting from a systems approach to molecular and cell biology. Application of single cell imaging techniques has demonstrated that individual cells in clonal populations may have very different phenotypes under the same environmental conditions [1] and that a pre-existing subpopulation of cells may survive a sudden environmental change that is lethal to the majority of cells, such as antibiotic treatment, thus gaining advantage [2]. These observations are particularly important in the context of survival strategies of bacterial pathogens. The phenotypic heterogeneity of isogenic bacterial populations has been implicated in the emergence of persistence and latent infection in *Mycobacterium tuberculosis* that makes this bacterium one of the most dangerous pathogens of mankind [3]–[5].

Phenotypic differences of genetically identical cells under the same environmental conditions have been attributed to the inherent stochasticity of biochemical processes [6]. According to theoretical predictions elementary chemical reactions involved in biochemical processes exhibit substantial stochastic fluctuations when low numbers of reactant molecules are involved within the small volume of a living cell. The existence of significant stochastic fluctuations in biochemical processes has been confirmed by numerous experiments including tracking of individual protein molecules in individual cells in gene expression processes [7]. The mechanism by which these fluctuations give rise to phenotypic diversity has been a subject of intensive study. In most cases phenotypic diversity has been attributed to stochastic fluctuations that result in switching between different stable states of the dynamical system occurring in a network that involves positive feedback loops [2], [8]–[10]. Alternatively, a network may exhibit excitable dynamics, where fluctuations can lead to transient excursions from a single stable state to an unstable, but slowly decaying, excited state [11], [12]. Yet another mechanism arises when a single stable state exists in the system, and the reaction network is effectively switched on and off according to the availability of one of the constituent chemical species [13], [14]. Here we describe a novel situation, in which a monostable or bistable two-component system supports a persistent approximate basal solution, owing to stochastic delays in the transcription of either histidine kinase or response regulator genes. However, once a particular cell has reached a fully induced level of gene expression there is a negligible chance that it will revert to the basal state.

Two-component signal transduction systems (TCS) are a very common mechanism by which bacteria sense external signals and induce the expression of genes that govern the response to environmental change. A particular environmental signal activates a specific membrane-bound histidine kinase (HK), which in turn activates its partner response regulator (RR) via phosphoryl donation. The response regulator itself activates the transcription of multiple genes whose products enable the bacterium's adaptive response to the change it has sensed. A common experimental design is to introduce a reporter gene whose transcription is controlled by the response regulator, and to monitor the TCS output by measuring the number of reporter protein molecules produced by the reporter gene. We shall do the same in the numerical and analytical studies we present in this paper. We will later consider two scenarios: autoregulation of the RR gene, where RR activates its own transcription and so positive feedback is present, and the constitutively expressed RR gene, where activated transcription of RR is absent. It has already been shown that stochastic fluctuations in the expression of RR and HK genes lead to population heterogeneity with respect to the expression level of genes regulated by the TCS. Sureka et al. [3] used flow cytometry to show that the MprA/MprB TCS in *Mycobacterium smegmatis* leads to heterogeneous activation of the stringent response regulator Rel. that permits persistence to develop in *Mycobacterium tuberculosis* [4], [5]. Sureka et al. complemented their experimental observations with numerical simulations of a stochastic kinetic model of the TCS, demonstrating that autoregulation of the RR results in bistable behaviour and that stochastic fluctuations in gene expression switch the system between the two stable states corresponding to two different phenotypes. Zhou et al. [7] had earlier used flow cytometry to measure gene expression in single *Escherichia coli* cells from a genetically identical population, in order to study cross-activation of the RR PhoB by noncognate HKs in the PhoR/PhoB TCS, and found a bimodal pattern of fluorescent protein reporter gene expression. Subsequently, Kierzek et al. [15] built the most comprehensive stochastic kinetic model of two-component system signalling published to date and used data of Zhou et al. to show that their model reproduces flow cytometry distributions of TCS-regulated fluorescent protein reporter gene expression. Further computer simulations demonstrated two response modes of the TCS leading to population heterogeneity. In the ‘all-or-none’ response that arises when the RR gene is positively autoregulated, the reporter gene is expressed either at fully induced or at basal level, and a change in the external signal strength results in a corresponding change in the fractions of cells expressing the gene at basal and fully induced level. Alternatively, population heterogeneity can be observed in a ‘mixed mode’ that occurs when the RR gene is constitutively expressed. In this response mode one population of cells expresses the gene at basal level, while in another cell population the gene is expressed at a level that depends on the signal strength. The mixed mode thus combines features of all-or-none and graded responses.

In this work we use deterministic, probabilistic and equation-free methods to analyse the potential for simultaneous coexistence of different phenotypes in the Kierzek, Zhou and Wanner stochastic kinetic model of TCS signalling [15] (hereafter KZW). The application of equation-free methods to biochemical reaction networks has typically focused on simple models of small networks [16], [17], though there have been some studies of larger scale networks [18]–[20]. Here we apply them for the first time, to the best of our knowledge, to a detailed model of signal transduction processes. Our results show that population heterogeneity can be generated by a molecular interaction network even when it is not multistationary. A deterministic bifurcation analysis of reaction rate equations derived from the KZW stochastic kinetic model shows that the mixed mode is absent in this framework. However, an equation-free analysis of the stochastic model, using the Gillespie algorithm with tau-leaping as a black-box time-stepper, in order to find stationary states for the mean of an ensemble of stochastic trajectories, reveals the long-term persistence of an approximate basal solution that combines with the graded response to produce the mixed mode. This confirms the results of a probabilistic analysis that establishes the essential stochastic nature of the mixed mode. The same techniques also show that stochasticity results in the observation of the all-or-none bistable response over a much wider range of external signals than would be expected on deterministic grounds. In summary, our work uses a detailed mechanistic model of the major signal transduction and gene regulation mechanism to show that multistationarity and positive feedback are not necessary for the emergence of phenotypic diversity and that deterministic bifurcation analysis is not always sufficient to explain phenotypic switching.

In the Results section we first introduce the stochastic kinetic model that we shall be analysing, then we analyse the deterministic reaction rate equations that govern the chemical concentrations in the thermodynamic limit, and show that these do not permit a mixed-mode solution. In the following subsection we analyse the discrete stochastic system using equations for the expected (probabilistic mean) number of molecules of each chemical species present, and show that slow transcription of either or both of the histidine kinase or response regulator genes can lead to persistence of reporter gene expression at a level that is approximately basal when it would not be expected on deterministic grounds. In the final subsection of Results we show that equation-free methods can locate this unexpected basal expression solution and investigate its stability using only direct stochastic simulations. Thus we confirm the findings of our probabilistic analysis, and also demonstrate the potential of equation-free methods to shed light on stochastic effects in large complex systems where a probabilistic analysis is too difficult to perform. In the Discussion we summarise our findings and highlight their biological significance. The Methods section includes mathematical details of the probabilistic and equation-free analyses.

## Results

### Stochastic kinetic model

**Figure 1:**
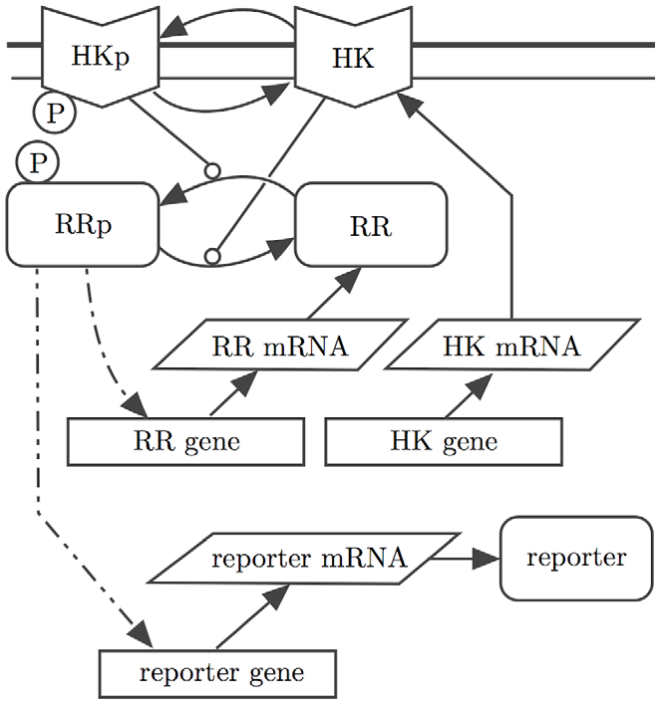
Simplified diagram of the KZW stochastic TCS model. (After [15].)

We base our stochastic kinetic model of the PhoBR TCS in *E. coli* on that of Kierzek, Zhou and Wanner [15], summarised in Fig. 1. (A detailed representation of the model in Systems Biology Graphical Notation (SBGN) is given in [15].) We are interested in stochastic switching of reporter gene expression, and hence in the numbers of reporter protein molecules produced. The external signal is modelled as the ratio of the HK autophosphorylation to dephosphorylation rates. Dashed arrows on the diagram indicate activated transcription of the response regulator and reporter genes, modelled using the Shea-Ackers formalism [21], where the reaction rate increases with the concentration of phosphorylated RR, saturating for large [RRP] at a level much higher than in the absence of RRP. As mentioned above, we will consider two cases: the autoregulated and the constitutively expressed RR gene. Transcription and translation of the response regulator, histidine kinase and reporter genes are modelled as pseudo-first-order reactions. The circle-headed arrows indicate HK/RR complexes in phosphate transfer processes, according to the Batchelor & Goulian model [22]. Included in our model but not shown in the diagram are dimer formation and dissociation and also reporter protein and mRNA degradation.

KZW simulated the reaction network using the Gillespie algorithm [23] for direct stochastic simulation, and incorporating gene replication and cell division events. The Gillepsie algorithm updates the number of molecules 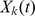 of the 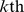 chemical species, using the propensity functions 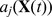, where 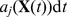 is the probability that the 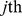 reaction takes place in the time interval 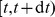, and its associated stochiometric vector 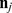 whose 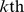 component is the change in 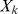 caused by the 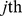 reaction. The propensity functions for the reactions involved in the KZW model are given in Table 1, where 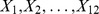 are the numbers of molecules of phosphorylated RR protein (RRP), mRNA of RR (mRNA-RR), RR protein (RR), HK protein (HK), phosphorylated HK dimer (HK2P), complex of RR and phosphorylated HK dimer (RR-HK2P), complex of phosphorylated RR and HK dimer (RRP-HK2), mRNA of reporter (mRNA-Rep), reporter protein (Rep), mRNA of HK (mRNA-HK), phosphorylated RR dimer (RR2P) and HK dimer (HK2) respectively, and 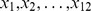 are the corresponding concentrations. The correspondence between chemical species and model variables is also given in Table 2 for ease of reference. The parameters 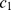 to 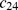 given in Table 1 were chosen by KZW to accord with experimental data where available, or with validated models of prokaryotic gene expression or, in cases where it did not affect the qualitative results, they were chosen at will [15]. The concentration of RNA polymerase (RNAP) is fixed, at 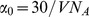, in order to model transcription and translation as pseudo-first-order reactions, following KZW [15], where 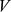 is the cell volume and 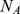 is the Avogadro constant and we set 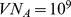. The concentrations of the various degradation products mentioned in Table 1 do not influence the propensity functions and so we do not include them as variables in our model. The external signal is modelled as the ratio of the autophosphorylation to dephosphorylation rates for histidine kinase, 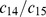, which we vary by keeping 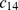 fixed and changing 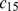.

**Table 1:** Chemical reactions in the PhoBR *E. coli* stochastic kinetic model [15].

Description	Reaction	Propensity function	Stochastic rate constant ( 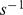 )
Activated transcription of RR	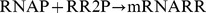	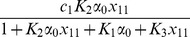	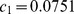 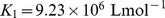 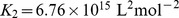 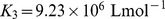
Basal transcription of RR	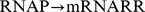	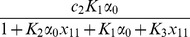	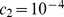
Translation of RR	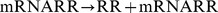	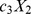	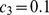
Degradation of RR mRNA	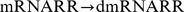	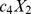	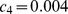
Degradation of RR and variants	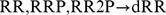	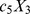 , 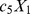 , 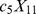	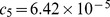
Transcription of HK	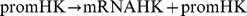	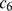	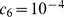
Translation of HK	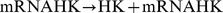	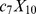	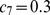
Degradation of HK mRNA	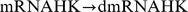	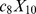	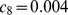
Degradation of HK and variants	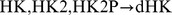	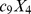 , 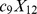 , 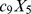	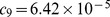
Binding of RR to phosphorylated HK	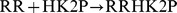	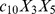	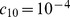
Phosphorylation of RR	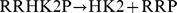	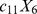	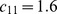
Binding of unphosphorylated RR to HK	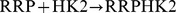	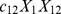	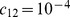
Dephosphorylation of RR	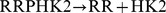	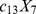	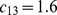
Autophosphorylation of HK	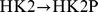	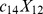	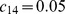
Dephosphorylation of HK	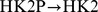	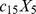	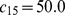 (varies)
Activated transcription of reporter gene	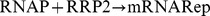	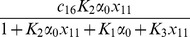	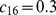
Basal transcription of reporter gene	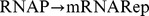	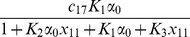	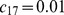
Translation of reporter gene	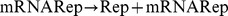	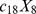	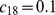
Degradation of reporter transcript	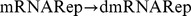	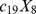	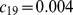
Degradation of reporter	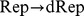	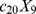	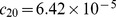
Dissociation of HK dimer	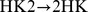	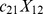	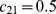
Association of HK dimer	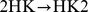	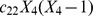	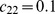
Dissociation of phosphorylated RR dimer	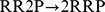	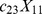	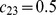
Association of phosphorylated RR dimer	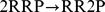	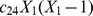	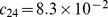

Reactions included in the KZW stochastic model [15], together with the associated propensity functions and values of the stochastic rate constants. Values given are for the autoregulated, slow transcription case. The value of 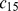 varies with the external signal in our simulations. d-mRNA-RR, d-RR, d-mRNA-HK, d-HK, d-mRNA-Rep and d-Rep are degradation products of mRNA-RR, RR, mRNA-HK, HK, mRNA-Rep and Rep respectively. prom-HK is the promoter region of the HK gene.

**Table 2:** Correspondence between chemical species and model variables.

Chemical species	Short name	Concentration	Number of molecules
Phosphorylated response regulator protein	RRP	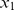	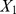
mRNA of response regulator	mRNA-RR	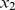	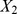
Response regulator protein	RR	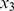	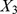
Histidine kinase protein	HK	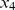	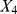
Phosphorylated histidine kinase dimer	HK2P	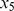	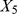
Complex of RR and phosphorylated HK dimer	RR-HK2P	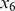	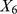
Complex of phosphorylated RR and HK dimer	RRP-HK2	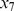	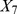
mRNA of reporter	mRNA-Rep	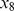	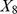
Reporter protein	Rep	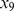	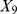
mRNA of histidine kinase	mRNA-HK	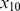	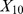
Phosphorylated response regulator dimer	RR2P	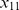	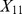
Histidine kinase dimer	HK2	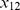	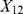

In summary, the Gillepsie algorithm consists of randomly selecting the next reaction that occurs to be 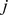 with probability proportional to 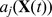, and randomly selecting the time, 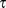, until that next reaction takes place from an exponential distribution with rate parameter 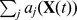. The vector 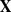 is updated according to the numbers of molecules created and consumed in reaction 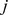, and time is increased by 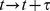 [24]. Stepping forward in time in this way gives a single realisation of the system. Typically, many realisations are computed to give a fuller picture of the system behaviour. KZW started each realisation at 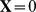 at time 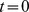, and performed 10,000 realisations, each of 20,000 s duration, for each parameter combination of interest.

KZW were interested in two sets of comparisons: autoregulation of the RR gene versus constitutive expression, as discussed above, and fast versus slow transcription of HK. KZW chose an operating point for their system such that the mean steady state numbers of RR and HK protein molecules were 3800 and 25 respectively. The parameter values given in Table 1 are those for the autoregulated, slow transcription case. To simulate a constitutively expressed response regulator gene, we break the feedback loop by replacing the first two response regulator transcription reactions in Table 1 by the reaction 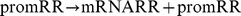, where prom-RR is the promoter region of the RR gene, with propensity function 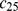, where the rate constant 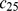 is chosen to lead to the same system operating point in order to permit fair comparison with the autoregulated case. KZW found that a value of 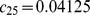 accomplished this [15]. In order to isolate the effect of variability in HK expression, KZW fixed the overall rate of transcription followed by translation to be 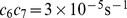. In the slow transcription, fast translation case the rate constants were 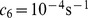 and 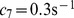, while in the fast transcription, slow translation case these values were swapped. Slow transcription followed by fast translation produces HK in bursts, while fast transcription and slow translation leads to more continuous production [15].

**Figure 2:**
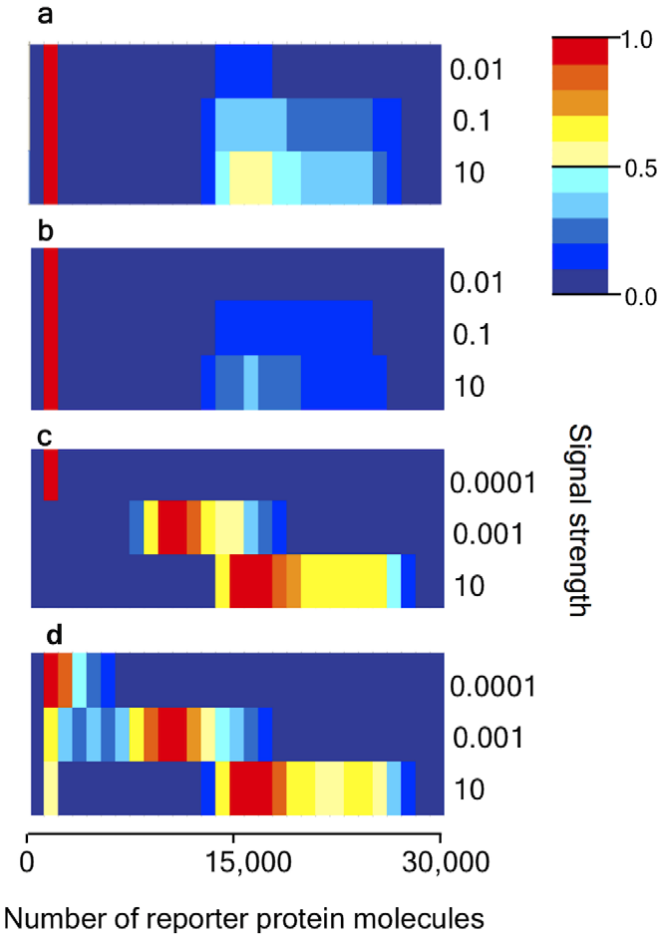
Results of Kierzek, Zhou and Wanner [15]. Histograms of 10,000 realisations at time 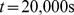, as fractions of largest value: for a) autoregulated RR gene & fast transcription of HK; b) autoregulated RR gene & slow transcription of HK; c) constitutively expressed RR gene & fast transcription of HK; d) constitutively expressed RR gene & slow transcription of HK. (Adapted from [15]. Reproduced by permission of The Royal Society of Chemistry http://pubs.rsc.org/en/content/articlelanding/2010/mb/b906951h DOI: 10.1039/B906951H.

With autoregulation of the RR gene and fast transcription of HK (Fig. 2a) KZW saw stochastic switching between the basal and fully induced levels of reporter gene expression - a so-called ‘all or none’ response. In other words, some trajectories showed very little reporter protein present at time 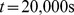, while some showed a large amount, and the number of reporter protein molecules produced during the productive trajectories did not seem to depend strongly on the external signal strength. The picture was similar with autoregulation and slow HK transcription, but there were fewer realisations at the activated level (Fig. 2b). In the case of a constitutively expressed RR gene and fast HK transcription, there was no stochastic switching - a graded response was seen instead, where the number of reporter protein molecules produced increased with increasing signal strength (Fig. 2c). An interesting novel case was found when the RR gene was constitutive, but transcription was slow, when stochastic switching and a graded response were seen simultaneously - a so-called ‘mixed mode’ (Fig. 2d). It is the unexpected existence of this mixed mode that we seek to explain through our analyses below.

### Reaction rate equations and deterministic bifurcation analysis

In the thermodynamic limit where the cell volume and the numbers of molecules of each chemical species tend to infinity, but the concentration of each species remains constant [24], the KZW model for the system containing an autoregulated RR gene can be reduced to the following set of deterministic reaction rate equations that describe mass-action kinetics for continuous real-valued concentrations:
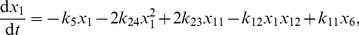
(1)

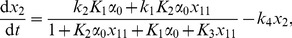
(2)

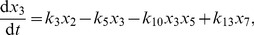
(3)

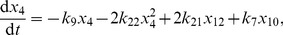
(4)

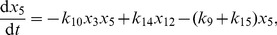
(5)

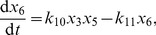
(6)

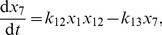
(7)

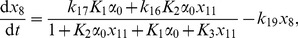
(8)

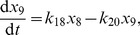
(9)

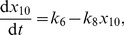
(10)

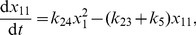
(11)

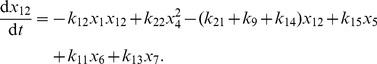
(12)


If the RR gene is constitutively expressed, then equation (2) becomes
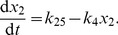
(13)


The deterministic rate constants are appropriately scaled versions [24] of those used in the stochastic kinetic model: 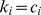 for 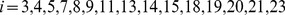, 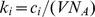 for 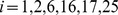, 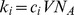 for 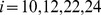.

In reality for this system some species remain low in number, fluctuating between zero and a small integer number. Thus we expect the deterministic continuous analysis based on these equations to give clues as to the system behaviour, but to fail to describe it adequately in some important respects.

Note that equations (8) and (9) decouple from the rest of the system, being dependent only on the input value of 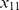, but not feeding back into the remaining equations through the values of 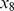 and 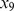. Thus the reporter protein concentration, 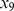, is ultimately determined by that of the phosphorylated RR dimer, 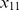.

We considered four sets of parameter values that gave every combination of fast and slow transcription with autoregulated and constitutively expressed RR gene. In each case we first found a stationary solution of the reaction rate equations for a particular value of the external signal (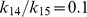) numerically and then continued it over a range of external signals 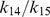, where 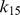 was varied, using the XPPAUT software package [25] to produce a deterministic bifurcation diagram.

In the autoregulated case, the basal level of reporter gene expression is shown at zero external signal (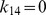), where it corresponds to the following fixed point of equations (1)–(12):
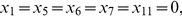
(14)

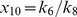
(15)

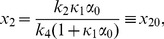
(16)

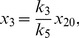
(17)

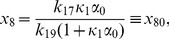
(18)

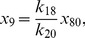
(19)

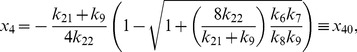
(20)

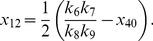
(21)


More generally it can be shown that the fixed points of equations (1)–(12) are given by
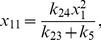
(22)

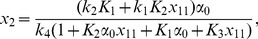
(23)

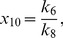
(24)

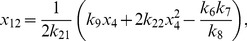
(25)

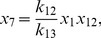
(26)

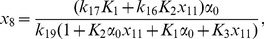
(27)

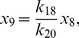
(28)

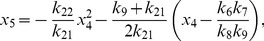
(29)

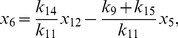
(30)

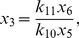
(31)where 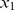 and 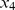 are the solutions of the nonlinear equations
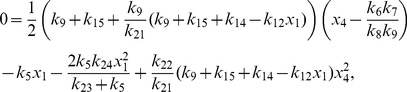
(32)

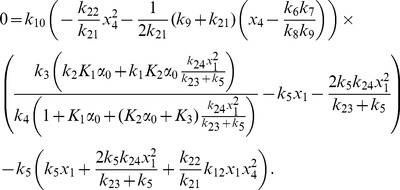
(33)


In the constitutive case, equations (16) and (23) become 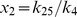 and equation (33) becomes
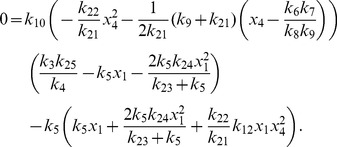
(34)


**Figure 3:**
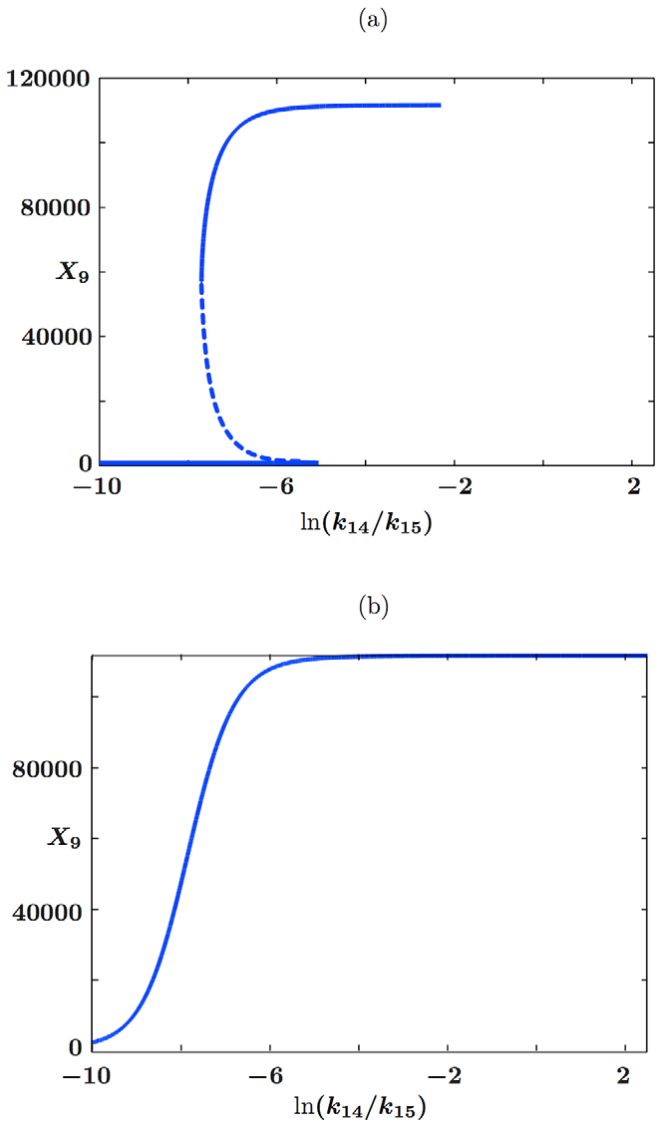
Bifurcation diagrams for the deterministic reaction rate equations. The diagrams are constructed using XPPAUT for equations (1)–(13) and the parameter values given in Results. Numbers of reporter protein molecules produced are plotted against the natural logarithm of the external signal 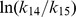, in the a) autoregulated and b) constitutive cases, showing a bistable and graded response respectively. Bold lines denote stable solutions and dashed lines denote unstable solutions.

It is clear that, apart from the value of 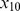, the steady solutions depend on the rates of HK translation and transcription only through the product 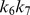, which we have set to be constant. Thus the slow and fast transcription cases have the same fixed points in the deterministic framework. It turns out that these fixed points also have the same stability type over the range of external signals that we examined, and so the deterministic bifurcation diagrams are the same for fast and slow transcription. The autoregulated case (Fig. 3a) shows a classical bistable scenario, with a stable state corresponding to the basal level of expression of reporter protein, coexisting over a range of external signals (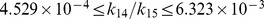), with a stable state corresponding to the activated level of expression. For 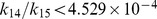 only the basal expression solution exists, while for 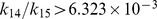 only the activated state is possible. The switch between these two states, gives a classical ‘all-or-none’ response: in a population of cells, for a given external signal, some will show activated expression of reporter protein and some will show only basal level expression. This case corresponds to Figs. 2a (fast transcription) and 2b (slow transcription) of the KZW results, where an ‘all-or-none’ response is indeed seen. On breaking the feedback loop to investigate the constitutively expressed RR gene, a graded response is seen (Fig. 3b), where the amount of reporter protein produced rises steadily as the external signal is increased, and this solution is stable. Both Figs. 2c and d show KZW results using a constitutively expressed RR gene, but while they saw a graded response in the fast transcription case (Fig. 2c) they saw a mixed mode when transcription was slow (Fig. 2d), where the basal level of reporter gene expression persists for at least 20,000 s in some cells even for quite large external signals. In our deterministic bifurcation analysis, this basal solution is absent and so there is no mixed mode. We deduce that the mixed mode results from stochasticity and/or discreteness.

### Analysis of the discrete stochastic model

We want to find the approximate steady state of the discrete stochastic system. It does not have true fixed points, such that 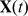 remains constant for all time. However, we can look for fixed points of the expected value, or mean, 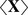. As shown in Methods, 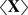 evolves according to the differential equation
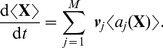
(35)


This is different from the reaction rate equations for the evolution of the vector of concentrations 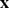 because 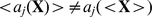 in general for nonlinear 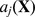 [24]. Thus when the RR gene is autoregulated, the rates of change of the components 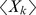 of the mean are
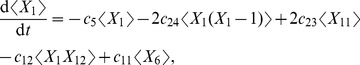
(36)

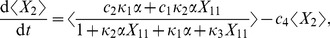
(37)

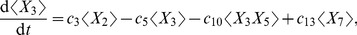
(38)

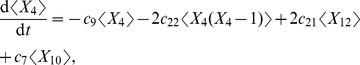
(39)

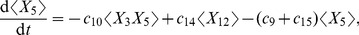
(40)

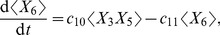
(41)

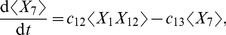
(42)

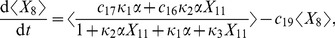
(43)

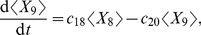
(44)

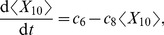
(45)

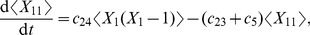
(46)

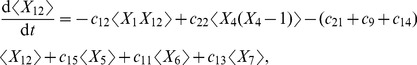
(47)where 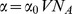, 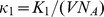, 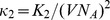 and 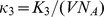. If the RR gene is constitutively expressed, then equation (37) becomes
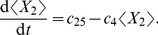
(48)


To look for a basal solution for these equations, we set 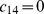 for a zero external signal, and look for solutions 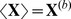 such that 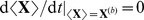. In Methods, we show that 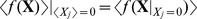. Bearing this in mind, we find that the basal solution for the means satisfies 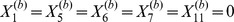,
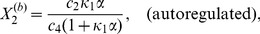
(49)

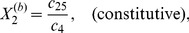
(50)

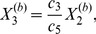
(51)

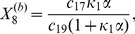
(52)

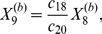
(53)

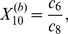
(54)and that 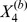 and 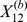 correspond to fixed points (if such can be found) of the equations
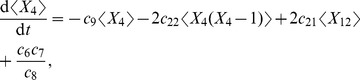
(55)

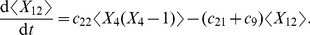
(56)


These last two equations are not in closed form, involving the higher-order moment 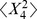, and so we cannot deduce from them whether a solution for 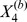 and 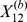 actually exists. If the basal solution does exist, then we see that, with the exception of the values of 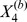 and 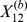 it is the equivalent of the deterministic basal solution with the deterministic rate constants replaced by their stochastic equivalents.

In order to understand the stochastic behaviour, the equations for the evolution of the mean are not sufficient. For a given realisation of the system, the 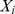 must take non-negative integer values, and this discreteness turns out to be important in understanding the existence of basal solutions where they are not predicted by the mass-action or mean reaction rate equations. We have 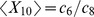 from equation (45). In the case of slow transcription, where 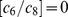 (and where here and hereafter 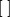 indicates the rounded value, with half integers being rounded upwards), the closest an individual trajectory can get to the fixed point of the mean is at 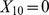. We can now look for fixed points of 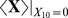, which satisfy equations (36)–(44), (46) and (47), with the term involving 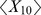 being zero in equation (39). In fact, in the slow transcription case we have 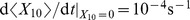 and so if we can find a steady state for the remaining components of 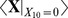, we would expect it to persist over a timescale of approximately 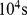.

Equations (43) and (44) show that the basal level of reporter protein production occurs when 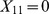, in other words when no phosphorylated HK dimer (HK2P) is present, so we will look for a steady state solution 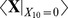 that also has 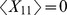 and call it 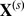. Thus we have 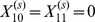, and we now look for values of the remaining components of 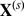 that are consistent with this. We are no longer restricting the external signal, 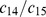, to be zero. However, we find 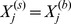 for all 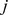 except 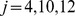 for which 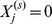. Thus an approximate steady state 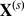 can be found that corresponds to a basal level of reporter protein production for arbitrary values of the external signal.

For the parameter values used in our study, we find that in the autoregulated slow HK transcription case 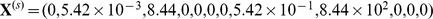 and in the constitutive slow transcription case 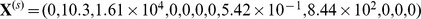. In both autoregulated and constitutive cases, these solutions are equivalent to the deterministic and mean basal solutions except for the values of 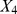, 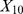 and 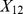. Thus we expect to see the basal level of reporter protein in a proportion of cells for **all** values of the external signal in the slow transcription case for both the constitutively expressed and autoregulated RR gene. In the constitutive case, this is the origin of the mixed mode, and in the autoregulated case it is why the basal solution is seen at unexpectedly high values of the external signal.

Note from equation (36) that a requirement for the existence of an approximate discrete basal steady state with 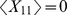 is that 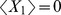 and equation (46) shows that this in turn requires that there be no RR-HK2P complex present (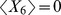). Equation (6) then implies we must have 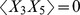. This holds for the majority of trajectories over long times for both slow transcription cases, since slow transcription of HK means that the levels of mRNA-HK is typically zero (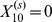), and when that is true we can find steady states where there is no HK protein (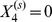) or HK dimer (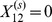) and hence no phosphorylated HK dimer forms (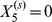), as we have just shown.

In the autoregulated cases, if we look for steady state solutions for the mean that have 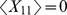, then from equations (37) and (74) we have
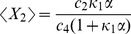
(57)and for our parameter values this gives 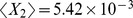, which indicates that the average number of mRNA-RR molecules present is very low and transcription of RR is slow. The closest an individual trajectory can come to this value is at 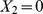, so we look for fixed points 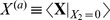, with 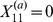, that satisfy equations (36) and (38)–(47) with the left-hand sides equal to zero. If we can find such a solution, we would expect it to persist over timescales of about 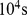 (since 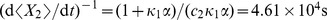 at 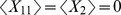). Since 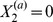 (no mMRNA-RR is present), and since for a basal solution we also have no RR2P (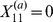) or RRP (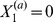) and thus no RRP-HK2 (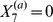), we find from equation (38) that it is consistent to have no response regulator protein (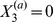) and so again 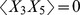 is satisfied. Thus in the autoregulated, fast HK transcription case we find the approximate basal solution 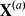 such that 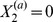, 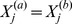 for 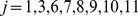, and 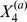, 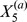 and 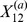 correspond to fixed points of the equations
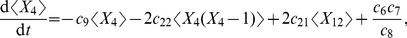
(58)

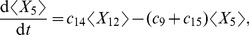
(59)

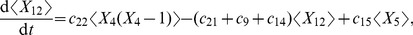
(60)if they exist. (Again these equations involve the second order moment 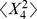, and so we cannot deduce from them the existence of a fixed point of the mean.)

For the parameter values of our study this gives 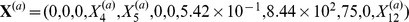 for the autoregulated fast transcription case. Although the terms in 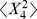 prevent us from determining 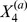, 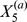 and 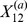 explicitly, we see that
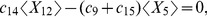
(61)

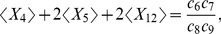
(62)and hence, assuming that the solution does indeed exist, we must have
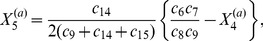
(63)

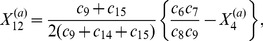
(64)where 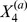 is chosen such that
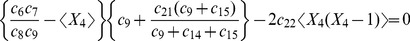
(65)holds.

Since 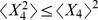, 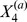 must satisfy
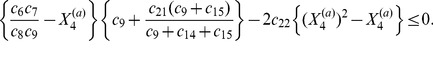
(66)


For the parameter values just mentioned this gives 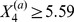, and from equations (63) and (64) we then see that 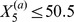 and 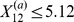. Since 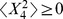, we must also have
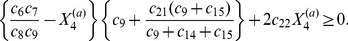
(67)


This is automatically satisfied if 
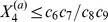
, which is required if equations (63) and (64) are to have non-negative solutions for 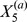 and 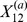.

Note that in the autoregulated, slow HK transcription case, we can find an approximate basal solution 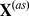 that has both 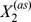 and 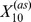 equal to zero: in other words 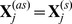 for 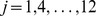 and 
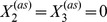
, with the growth rates of all components being zero except for 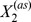 and 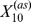 where the growth rates are 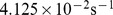 and 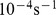 respectively. For the parameter values of our study we have 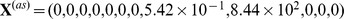.

In the case of the constitutively expressed RR gene with fast HK transcription, no approximate basal steady state can be found. The rapid production of mRNA-HK (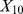) in the fast transcription cases - a steady state of approximately 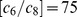 molecules from equation (45) - ultimately leads to the production of phosphorylated HK dimer (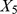). The rate constant, 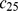, for the constitutively expressed RR gene is chosen to produce similar numbers of reporter protein molecules to those found in an activated cell in the autoregulated case. Thus, when phosphorylated RR dimer (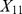) is scarce, RR transcription is much faster for the constitutively expressed than autoregulated gene. Equation (48) gives a steady state of approximately 10 mRNA-RR molecules in the constitutive cases, since 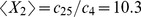, and hence RR protein (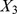) is also present at high levels. The combination of both phosphorylated HK dimer and RR protein allows RR-HK2P complex (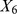) and hence RR2P (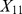) to form and ultimately leads to the presence of reporter protein (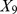) at levels much higher than basal.

Starting from the approximate basal solutions, we need RR protein (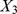), and prior to this mRNA of RR (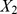), and HK2P (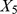), and prior to this HK protein (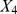) to form before reporter protein can be formed. This will happen only very rarely because either 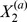 and 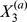 are zero (autoregulated cases) or 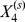 and 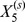 are (slow HK transcription cases) or both (autoregulated slow transcription case) and the corresponding growth rates are tiny or zero, showing that the reactions involving these species are well-balanced at 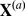, 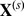 and 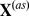. Thus the approximate basal solution is expected to persist over long times for a significant proportion of trajectories, or equivalently in a significant proportion of cells. Only in the constitutive fast HK transcription case is there the required combination of nonzero 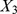 (and 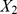) and 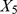 (and 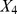) to cause the production of RR-HK2P complex (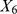) and lead within a short time for the vast majority of trajectories (or cells) to the presence of reporter protein (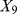) at levels above basal. This is the only case in which the basal solution is not observed for high external signals, as can be seen from Fig. 2c. Only a graded response is observed.

The results show that slow transcription of either or both of the HK and RR genes can lead to the persistence of the basal solution where it would not be expected from analysis of the deterministic reaction rate equations. The discrepancy between the deterministic and discrete stochastic models arises from the fact that trajectories do not remain close to the basal level of expression for all time in the stochastic model when the basal solution is not a stable fixed point of the system. Rather they eventually approach the discrete stochastic equivalent of the steady-state solution found in the deterministic model. However, there is a delay before transcription of HK and RR is initiated during which a near zero level of expression is observed. HK transcription takes place at a (stochastic) rate 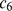 to give mRNA-HK, which is then translated at a rate 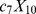, where 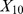 is the number of mRNA-HK molecules. For fixed 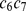, if the transcription rate constant 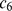 is small, transcription occurs in bursts [15]: it is delayed for a long time in some realisations, followed by very rapid translation when the number of reporter protein molecules climbs up quite quickly towards its steady state value. Hence a basal level of expression is observed for a long time in some realisations of the discrete stochastic model. This is the origin of the mixed mode observed in the constitutive case (Fig. 2d) for slow HK transcription initiation. On the other hand if transcription is initiated rapidly, corresponding to 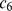 large, the number of mRNA-HK molecules rises quickly and production of reporter protein occurs more steadily as long as RR is also being transcribed fast enough; thus trajectories depart from the basal solution earlier on average. The basal expression level is therefore not observed over long periods (Fig. 2c). Note that the overall rate of transcription and translation of HK is the **same** in both cases, namely 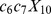. If RR is transcribed slowly then this can also result in the basal expression level being observed over long periods, even if HK is transcribed rapidly, and this is why we see a persistent basal solution in the fast HK transcription autoregulated case. Bistable behaviour of stochastic origin has also been found in direct stochastic simulations of autoregulated gene expression [13], [14], where although mRNA transcription and translation are either not considered, or treated as a single lumped step, stochastic activation of the gene by binding of a protein dimer is required before gene expression can proceed. However, in that case, while dimer binding is sporadic, the remaining biochemical reactions in the network are comparatively fast, so that gene expression is effectively switched on or off by the presence or absence of the dimer and thus proceeds in bursts. At any given time some cells in a population would be switched off and so a basal expression state would be found when it was not expected on deterministic grounds, but the mechanism is different from the one we see here, where a given cell may persist in a basal state over a long period before transitioning to a higher level of reporter gene expression.

The production of reporter protein at a level above basal, ultimately requires the simultaneous presence in the system of RR (response regulator protein, 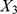) and HK2P (phosphorylated HK dimer, 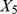). This is much more likely to happen if both are present in significant numbers, as is forced to occur by the forms of the mRNA-HK and mRNA-RR growth rates in the constitutive fast HK transcription case, than if either RR or mRNA-HK appears only sporadically, which is true for the former if response regulator is initially scarce and the gene is autoregulated and the latter if HK transcription is slow. In these cases, we expect reporter protein production to continue at basal level over long times. As the system is stochastic there will always be trajectories that do lead to production of reporter protein at much higher levels, and indeed every trajectory would be expected to reach these levels if we were to wait long enough, because eventually there would be a stochastic fluctuation large enough to bring the trajectory into the basin of attraction of the induced expression solution. Since bacteria have a finite lifetime we would in practice observe reporter protein production at induced expression levels in a proportion of cells and at basal levels in the remainder. In the autoregulated slow HK transcription case, both values 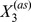 and 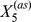 are zero, so it is to be expected that after a given time a smaller fraction of cells in this case produces reporter protein at induced levels than in the autoregulated fast HK transcription or constitutively expressed slow HK transcription cases, and it can be seen from Fig. 2 that this is indeed the case. In the constitutively regulated slow HK transcription case, the expected value of 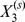 (response regulator protein) is very high at 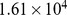, and so RR-HK2P complex (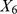) and hence reporter protein (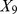) will be formed rapidly if stochastic fluctuations lead to the presence of a few HK2P molecules (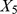). Thus after a fixed length of time, we expect a greater fraction of cells to show high levels of reporter protein in the constitutively regulated slow HK transcription case than in the fast HK transcription autoregulated case, where no more than about fifty HK2P molecules are present on average at steady state for the approximate basal solution (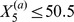), and so production of RR-HK2P complex (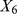) will proceed much more slowly when occasional molecules of response regulator (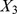) are formed. Again this confirms what is seen in Fig. 2.

### Equation-free determination of steady states for the stochastic kinetic model

In the previous subsection we analysed the equations for the time evolution of the mean 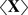 directly in order to find the approximate basal solutions that give rise to the mixed mode and to the extended range of signals over which an all-or-none response can be seen. We were fortunate in being able to do this: many reaction networks would be too complicated to succumb to this approach. However, it is possible to use direct stochastic simulations to gain information about the existence and stability of steady states of the probabilistic mean. In this subsection we use equation-free techniques to confirm the existence of the approximate basal solutions and investigate their stability. This approach could be extended to complex reaction networks that cannot be analysed explicitly.

So-called ‘equation-free’ methods (see [26]–[28] and references therein) are used to analyse the behaviour of dynamical systems that are either stochastic, or alternatively, deterministic of high dimension and with random initial conditions. The time evolution is obtained by a numerical time-stepping algorithm, and typically one is interested in characterising the asymptotic behaviour of the probability density functions of the associated state variables. Evolution equations for the probability distribution are often hard to write down in closed form, albeit their existence is guaranteed in most cases. However, ensembles of realisations of the dynamical system can be obtained by running the time stepper many times over for a given simulation time or time horizon, starting from a probability distribution of initial conditions. From these ensembles of realisations, moments (typically the mean and sometimes also the variance) of the probability distribution of state variables at the end time can be calculated. A key idea behind equation-free methods is that, if the high-order moments evolve much faster than (are *slaved* to) the low-order ones, there exists a closed evolution equation for the first few moments of the distribution. The method allows for the computation of steady states of, for example, the mean values of state variables, together with the corresponding Jacobian matrix that determines the stability eigenvalues for them, and so a bifurcation diagram can be constructed for these mean values. Thus all the powerful machinery of nonlinear dynamical systems can be brought to bear to explore systems for which explicit governing equations are not available.

Typically the equation-free method also encompasses the identification of fast and slow state variables and the use of ‘coarse projective iteration’ to speed up the time-stepping in large systems. We have not implemented these aspects here, in the first case because we did not expect any separation of variables into fast and slow to remain valid over the entire range of parameter regimes that we need to investigate, since we are explicitly varying the timescales of interest in this problem, and in the second case because the use of modified tau-leaping in the Gillespie algorithm performs a similar role to coarse projective iteration.

Equation-free methods have been demonstrated to work well for low-dimensional systems with tunable noise. They have also been used to examine stochastic simulations of (bio)chemical reaction networks in simple [16], [17], [29]–[31] and somewhat more complex cases [18]–[20]. Here we extend this work, by applying equation-free techniques to Gillespie algorithm simulations of a realistic biochemical reaction network of moderate complexity, which represents a significant computational challenge to the method.

In order to capture the purely stochastic near-zero solutions involved in the mixed mode (constitutive slow transcription case) and the extension of the basal expression level to high external signals in the autoregulated cases, we use an equation-free method [32] in which the Gillespie algorithm is a black-box time-stepper. We begin by identifying microscopic and macroscopic variables for the system. The microscopic variables are contained in the vector 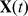, denoting the number of molecules of each species at time 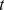. The coarse variables of our problem are then defined as an ensemble average of 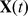 over a large number, 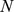, of realisations of the Gillespie algorithm
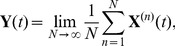
(68)where 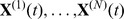 are the values of 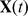 found in the realisations 1 to 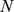.

A central role in the equation-free framework is played by the *coarse time-stepper*

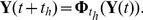
(69)


The operator 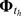 evolves the macroscopic state from time 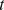 to time 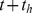 and, in general, is not available in closed form. However, it is possible to advance the coarse variables in time using independent microscopic runs of the Gillespie algorithm. The coarse time-stepper is then composed from these microscopic runs in three stages: lift, evolve and restrict [27] as described in Methods.

Once the coarse time-stepper is defined, we can find steady states 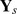 of the coarse evolution (69) by computing solutions to the equation
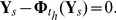
(70)


In our implementation, we find 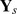 via Broyden's iterations: function evaluations consist in performing the lift-evolve-restrict steps mentioned above, whereas the Jacobian at points 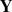 is determined numerically from the values of 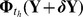 for various small perturbations of the mean 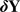. By choosing the time horizon, 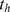, appropriately we can pick up metastable solutions that persist, on average, for that length of time, but are not true steady states of the system.

In practice, this turns out to be less straightforward than one might wish. The identification of a single fixed point requires hundreds of thousands to millions of realisations of the Gillespie algorithm (owing to the use of a large ensemble and the requirement for several iterations of the algorithm before convergence), and is consequently very slow, even when the calculations are parallelised. The error tolerance that can be achieved depends on the number of realisations in the ensemble, and so there is a trade-off between accuracy of the solution detected, the time horizon required and the practical feasibility of performing the calculation. Nevertheless, this method confirmed the insights described in the previous subsection. In the equation-free root-finding algorithm, we use 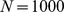 realisations and we set a relative tolerance of 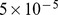 for Broyden's method. Finally, the time horizon 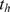 varies between 30 s and 500 s; as pointed out in [28], we expect the results to depend upon 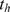. Note that in determining 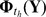 we use the value of 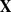 in each realisation that is computed at the last value of 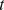 such that 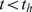. We typically observe convergence of the Broyden's method within 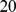 iterations, with the exception of a few points in the calculations of induced expression states where the solution jumps and the tolerance is met within 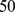 or 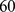 iterations. Since we are using a relative tolerance, our residuals never exceed 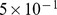, as the norm of our solutions is bounded by 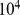.

Since the production of reporter protein, 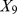, is controlled by the number of phosphorylated RR dimers present, 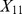, in this subsection we use the value of 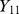, the mean value of 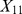, to illustrate our results. We first use the approximate basal solutions, 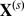, as an initial guess for the steady states at a very low value of the external signal in the constitutively expressed slow and fast transcription and autoregulated slow transcription cases, and use Broyden's method to find a nearby steady state. We then use this as a starting estimate of the solution at slightly higher external signal, converge once more to a nearby steady state, and in turn use this to find a solution at slightly higher external signal again. By this procedure of so-called ‘poor man's continuation’ we aim to trace out the dependence of the basal expression level of 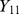 on the external signal. In the autoregulated fast transcription case, our initial guess at the lowest external signal level is 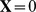.

**Figure 4:**
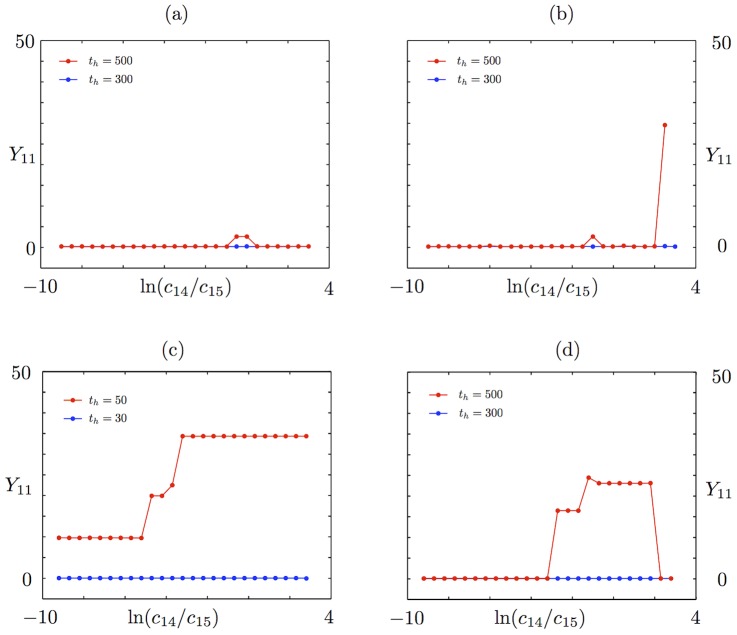
Timescales of persistence for approximate basal solution. Steady states are shown for the mean number of phosphorylated RR dimer molecules, 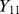, in equation (70), found using the equation-free numerical approach and poor man's continuation, starting from the lowest value of the external signal and an approximate basal solution or a zero solution and continuing towards higher signal values. The mean number of molecules produced is plotted against the natural logarithm of the external signal 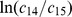 in the a) autoregulated fast transcription (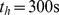 and 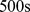), b) autoregulated slow transcription (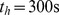 and 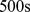), c) constitutively expressed fast transcription (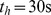 and 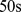) and d) constitutively expressed slow transcription (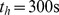 and 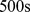) cases. Where the two curves coincide, the 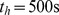 points are plotted and the 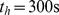 points are underlying.

Fig. 4 shows that with the exception of the constitutively expressed fast transcription case, a metastable basal solution with 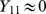 persists at all values of the external signal between 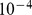 and 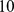 for a time horizon of 300 s. When the time horizon is increased to 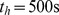 in both slow transcription cases, and in the autoregulated fast transcription case, we start to see the loss of this persistent basal solution at medium to large external signals (Figs. 4a, b and d). The 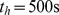 profile departs from zero for some values of the external signal, whereas the (underlying) 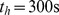 profile never does. We do not see a systematic variation of 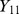 with external signal, because the level is still very low, and there is a certain variability in the numerical results that comes from using ensembles of stochastic trajectories. Thus, for example, no meaning should be attributed to the fact that the value of 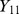 is zero in Fig. 4d for very high external signals, while it is nonzero for a range of signals below that: it is the fact that there are some nonzero values that is important. Furthermore, the use of poor man's continuation, where the last computed solution is used as an initial guess in the root-finding algorithm, means that we expect to see the same value of 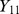 over a range of neighbouring values of the signal in this regime where we are looking at the first gradual loss of stability of the metastable basal solution. Once again, no meaning should be attributed to the clustering of values of 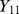 in this case.

In contrast to the other cases, the constitutively expressed fast HK transcription case only supports a basal solution for short time horizons: it is lost between 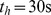 and 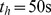 (Fig. 4c). This broadly supports the arguments in the previous subsection, where the basal solution was found to be absent in the constitutively expressed fast transcription case and to persist for approximately 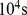 in the remaining cases. The fact that the basal solution persists at all in the first case results from the stochastic nature of the simulations: there will always be a short delay in the formation of reporter protein when necessary chemical species are initially absent. At 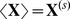, we have 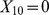 and 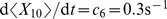. Since at least one molecule of mRNA-HK, 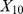, is needed to initiate the reaction sequence that leads to the production of phosphorylated RR dimer, 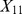, and hence an induced level of reporter protein, 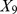, we expect the basal solution to persist for a time that is somewhat longer than 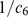, which in the constitutively expressed fast transcription case is approximately 3 s. The fact that the solution should persist for a somewhat longer time than 3 s results from stochastic delays in the formation of the intermediates 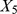 (phosphorylated HK dimer) and 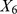 (RR-HK2P complex), which are also intially absent. This agrees reasonably well with the observed loss of the basal solution between 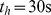 and 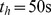. The loss of the basal solution at a high external signal at a time horizon of only 500 s in the remaining cases is a little surprising, but we postulate that the solution corresponding to induced expression of the reporter gene is strongly attracting at high external signals and so small fluctuations might be enough to move a sufficient number of individual trajectories into the basin of attraction of this higher solution branch so that a mean basal solution would no longer exist.

Once an average steady state is computed via Broyden's iterations, it is possible to calculate the corresponding Jacobian of the coarse time-stepper 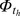 and infer the stability of the solution. Since the number of realisations used for the root-finding algorithm is relatively small (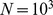) the resulting Jacobian computations are affected by noise. At selected points on the bifurcation curve, we increased the number of realisations to 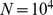 and repeated the Jacobian computations 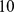 times.

**Figure 5:**
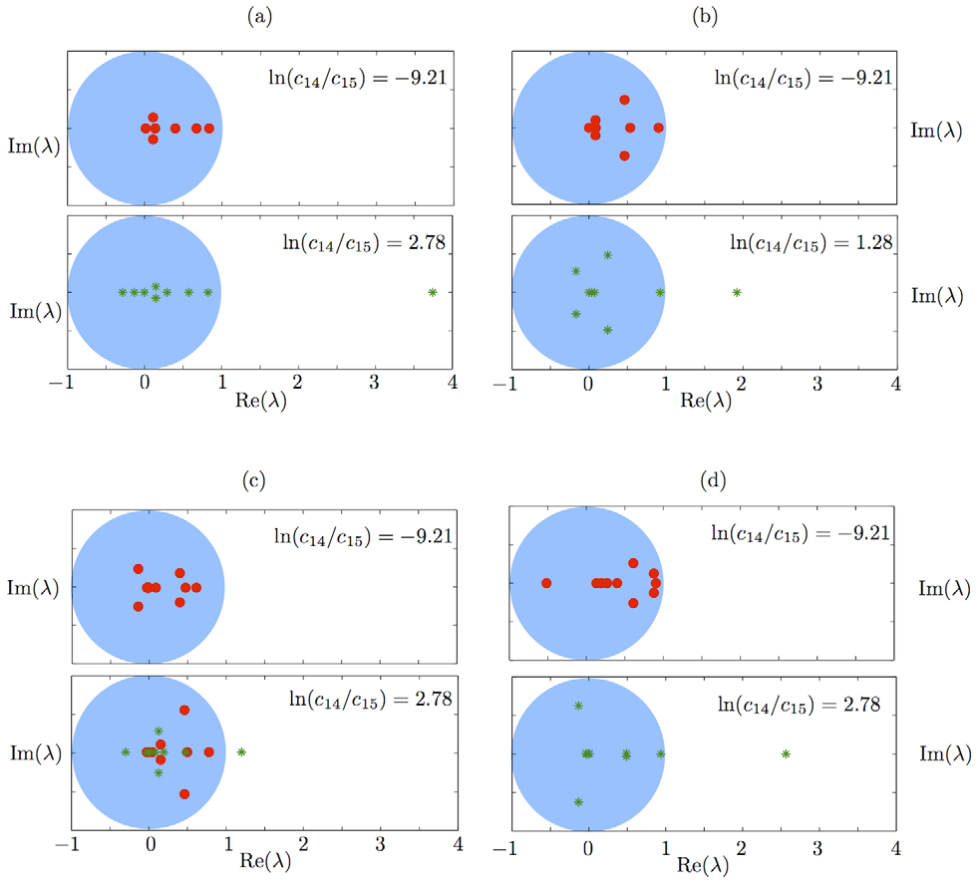
Stability of basal solutions. Spectrum of the Jacobian of the coarse time-stepper 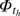 evaluated at basal solutions with low and high external signals for a) autoregulated fast transcription (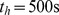), b) autoregulated slow transcription (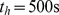), c) constitutively expressed fast transcription (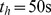) and d) constitutively expressed slow transcription (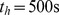) cases. Eigenvalues outside the unit circle (plotted in blue) indicate that the corresponding steady state is unstable. Eigenvalues corresponding to a spectrum that is stable overall are plotted as red circles. Eigenvalues corresponding to a spectrum that is unstable overall are plotted as green asterisks. In a), b), d) and the upper panel of c) one instance of the spectrum (of the 10 calculated) is shown. In the lower panel of c) two instances of the spectrum (of the 20 calculated) are shown: one stable and one unstable example.

In Fig. 5 we plot the spectra of the Jacobian evaluated at basal solutions for various values of the external signal 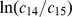. One instance (out of the 10 calculations) of each spectrum is plotted, except for the lower panel of Fig. 5c, where two instances are shown. In all four cases and for each of the 10 Jacobian computations, we found that solutions with low values of the external signal are stable. Conversely, high external signals lead to unstable steady states in the autoregulated fast and slow transcription cases and in the constitutively expressed slow transcription case. In the constitutively expressed fast transcription case we find a mixed picture for the high external signal: we repeated the Jacobian computation 20 times in this case and of those 11 gave a stable spectrum and 9 gave an unstable spectrum: one example of each is shown in the lower panel of Fig. 5c. We suggest that the difference in behaviour of the constitutively expressed fast transcription case compared to the other three may be due to the fact that the time horizon is much shorter - 50 s compared to 500 s - which could make the Jacobian calculations noisier, and that the steady state, which is effectively no longer a persistent basal solution, is further away from zero. Since the basal solution is expected to be only metastable at all values of the signal, we might have expected to see instability at low signals as well as high ones. However, in that region the higher solution branch - a true stable solution - lies close to the basal solution and so a) it may be hard to separate the two within our given error tolerance and b) the unstable eigenvalue of the basal solution will lie very close to the stability boundary and so we might classify it as stable within our given error tolerance. Broadly speaking a basal solution that appears stable at low external signals, but becomes unstable as the signal increases in strength, confirms our hypothesis of metastability.

**Figure 6:**
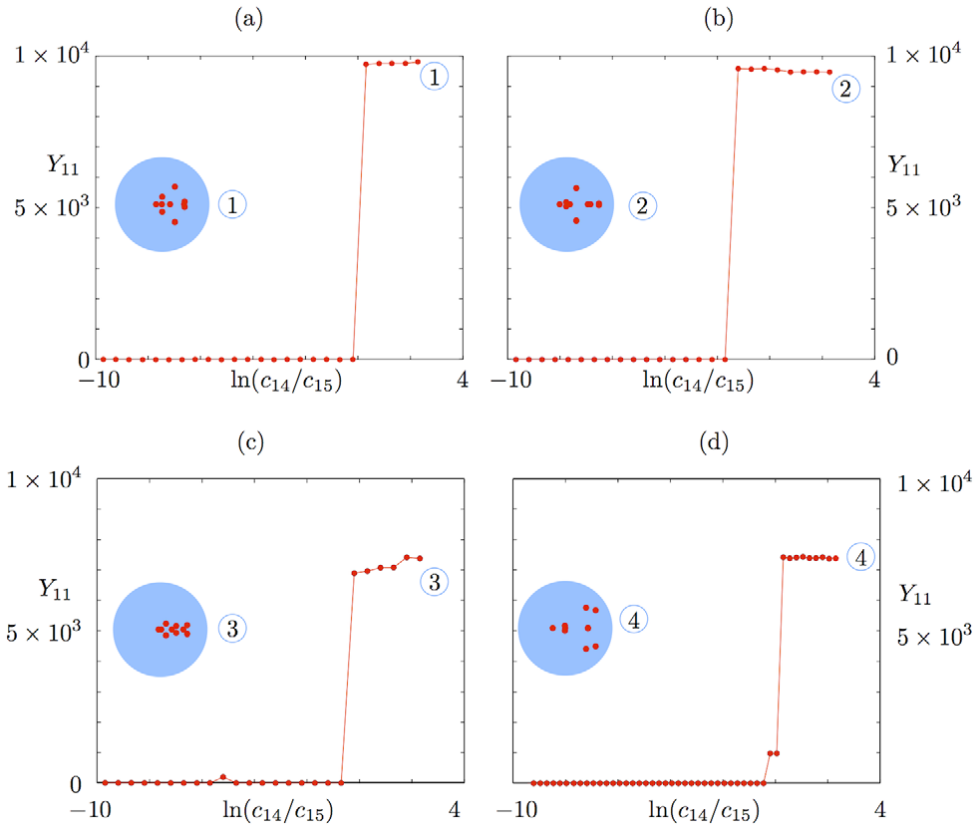
Equation-free tracking of induced expression states. Steady states are shown for the mean number of phosphorylated RR dimer molecules, 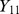, in equation (70), found using the equation-free numerical approach and poor man's continuation, starting from the highest value of the external signal and a point in the vicinity of the deterministic reaction rate solution and continuing towards lower signal values. The mean number of molecules produced is plotted against the natural logarithm of the external signal 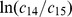 for time horizon 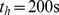 in the a) autoregulated fast transcription, b) autoregulated slow transcription, c) constitutively expressed fast transcription and d) constitutively expressed slow transcription cases. Spectra of stable steady states at the highest value of the external signal are plotted in the insets.

**Figure 7:**
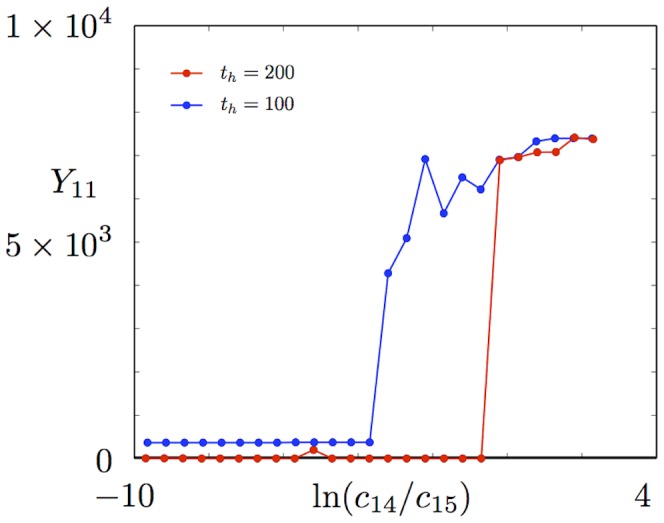
Graded response in the constitutively expressed fast transcription case. The equation-free tracking of the induced state in the constitutively expressed fast transcription case shows a graded response for 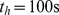 in one run. This behaviour is lost upon increasing the time horizon to 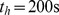 (see also Fig. 6c).

We can also investigate the existence of an induced expression solution using equation-free techniques. Here we start with a large external signal, and use a point in the vicinity of the solution predicted by the deterministic reaction rate equations (1)–(13) for induced expression of the reporter gene as an initial guess for a steady state. Once more we use poor man's continuation to follow the dependence of 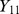 on the external signal, but this time tracking the solution as the external signal decreases. Any 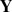 that we pick is likely to persist over sufficiently short time horizons, because we can pick a time interval so short that no reaction events are expected to take place. What we are really interested in are solutions 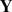 that persist over long time horizons. However, once 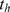 becomes greater than about 200 s, calculation times become so long as to be impractical. We would expect to find that for long enough 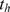, the autoregulated cases show ‘all-or-none’ behaviour where the activated expression solution suddenly vanishes below a threshold value of the external signal. In the language of nonlinear dynamics, this is a classical scenario of a subcritical bifurcation with hysteresis. By contrast for the constitutively expressed cases, we expect a smooth, graded, response as the external signal varies: in other words, a stable solution that grows in amplitude as the signal increases, but does not undergo a bifurcation. In the autoregulated cases, we do see an ‘all-or-none’ profile at the longest time horizon that we used, 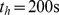 (Figs. 6a and b). However, we actually see similar behaviour in the constitutively expressed cases (Figs. 6c and d), though for the fast HK transcription case there is a hint of a graded response as the external signal decreases towards the point at which the basal solution appears. It is possible that the algorithm fails to converge on the induced steady state at intermediate values of the external signal, and instead locates the approximate basal solution. (Even in the constitutive fast transcription case, this solution may occasionally be found to persist for 200 s owing to the stochastic nature of the system, and since the root-finding algorithm is permitted quite a large number of iterations it may pick it up.) This may be because a larger ensemble of realisations is needed to achieve a given accuracy of solution as 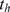 increases, as we describe below, but in practice using very large ensembles would have required infeasibly long run times. Interestingly we did find a graded response at 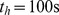 in the constitutively expressed fast transcription case, but we lost it for lower values of the external signal when we increased the time horizon to 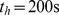 (see Fig. 7). Perhaps this is indeed owing to the decreased accuracy in locating the solution. However, we note that another run at 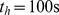 produced an ‘all-or-none’ profile (not shown) and that the autoregulated fast transcription case behaved similarly despite a graded solution not being expected there. Larger ensembles and longer run times would be necessary to resolve the question definitively. We have also computed the spectra corresponding to induced expression states for high values of the external signal, and found that they are stable in all cases (see insets in Fig. 6).

In order to calculate the steady states 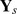, we repeatedly generate ensembles of realisations, each of which gives us a mean value 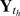. For a given 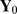, the variance of 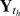 over a set of ensembles will be greater for longer 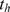 and smaller ensembles. Thus, as 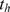 increases, we really should use a larger ensemble of realisations to allow us to determine steady states with sufficient accuracy. It is likely that this would allow us to distinguish better between the behaviour in the constitutively expressed and autoregulated cases, but in practice this is computationally prohibitive. Furthermore, as we approach a steady state, the time evolution of a given trajectory becomes very slow (because there is at least one growth-rate eigenvalue close to zero) and so extremely long time horizons would be needed to identify the location of the steady state accurately. Nonetheless we do pick up the basal expression state at low values of the external signal and the induced expression state at high signals in all four cases, thus demonstrating the ability of the equation-free method to locate metastable and stable solutions in complex reaction networks where explicit analysis cannot be used, but where the time evolution of the system is accessible through a numerical time-stepper.

## Discussion

We have sought to explain the existence of the mixed-mode response in a stochastic kinetic model of the PhoBR TCS in *E. coli*. We used bifurcation analysis to show that this mixed mode was absent in the framework of deterministic reaction rate equations that govern the concentrations of chemical species in the thermodynamic limit, and that it must therefore result from stochasticity in the discrete system. We then analysed the discrete stochastic system directly using equations for the probabilistic mean number of molecules of each chemical species present, and showed that slow transcription of either or both of the histidine kinase or response regulator genes can lead the reporter gene to be expressed at basal level in a fraction of cells within a population, even when the external signal is so high that this would not be expected on deterministic grounds. We confirmed this finding using equation-free techniques that located the unexpected persistent basal expression state and ascertained that it is unstable at high external signals. This persistence of the basal level of reporter gene expression is a truly stochastic phenomenon that arises because we must wait until random processes lead both RR protein and phosphorylated HK dimer to be present in the cell simultaneously so that the chain of reactions that lead to the production of reporter protein can proceed. The delay will be lengthy if either transcription process is very slow, and that is why a basal level of expression can be observed over long times. Combined with a graded response to the signal in the case where the RR gene is constitutively expressed, the persistent basal state leads to the ‘mixed-mode’ response described by KZW [15]. When the RR gene is autoregulated, the persistent basal state effectively extends the range of external signals over which an ‘all-or-none’ response can be seen.

These findings are important for understanding the survival strategies of bacterial pathogens. Two-component systems are the most prevalent mechanism of transmembrane signal transduction controlling gene expression programmes in bacteria [33]. Many of them are global regulators responsible for major switches in cell physiology. Thus stochasticity in the outcome of TCS regulation, that we have analysed in detail in this work, is likely to result in the coexistence of cells in qualitatively different physiological states. These cellular populations would inevitably respond differently to antibiotic treatment or immune system challenge and in many cases one of the populations would survive. Any global gene expression programme change leading to slow growth would slow down drug uptake and minimise the effects of drugs that block protein synthesis, and a change in the repertoire of surface proteins could enable a fraction of bacteria to survive an immune system attack. For example, a recent study shows involvement of the DosR response regulator in regulation of global metabolism and antibiotic response in *M. tuberculosis*. [34] Stochastic fluctuations in this particular TCS could therefore lead to the emergence of populations surviving antibiotic treatment.

While the role of TCS stochasticity in pathogen survival has already been recognised [3], the analysis of possible sources of phenotypic variation has been limited to autoregulated, bistable systems [3]. KZW did show numerical simulation trajectories exhibiting population diversity [15], but they did not analyse the mechanisms underpinning the observed phenomena in detail. In this work we demonstrate for the first time that a TCS that is not multistable can generate bacterial population diversity at the timescales relevant to bacterial responses. The parameter configuration for which this behaviour is observed in our model is biologically plausible. Among the number of two-component systems studied in detail, both autoregulated (e.g. PhoPQ in *E. coli* [35]) and constitutive cases (e.g. ArcAB in *E. coli* [36]) have been observed. According to quantitative measurements [37], the number of histidine kinase proteins present is low and could therefore be a source of stochastic fluctuations, as demonstrated in our model. In numerous two-component systems, such as ArcAB in *E. coli* [36], the HK gene is expressed from a different transcription unit than the RR gene and in these cases low expression levels can be regulated at both the transcription and translation levels. Therefore, observed TCS architectures and measured protein amount ranges show that two-component systems exhibiting population diversity in the absence of autoregulation and multistability are likely to exist. Moreover, the observed diversity of TCS architectures shows that the two-component system is a highly evolvable regulatory network motif. Depending on point mutations in the promoter and ribosome binding site (RBS), different modes of response to the external signal can be generated resulting in different distributions of phenotypic diversity in cellular populations. These mechanisms are likely to be subject to natural selection, especially in bacterial pathogens where population diversity conveys significant advantage. Our work shows for the first time that it is not only bistable two-component systems that are potential sources of phenotypic diversity in the evolution of bacterial populations. Thus experimental work on the stochasticity of two-component systems should not focus exclusively on multistable, autoregulated systems as has been the case so far. The analysis we have presented indicates that one should also consider the case where RR and HK genes are not autoregulated through positive feedback and where transcription of the HK gene is not coupled to the RR gene in an operon structure. Our study predicts that mutations in the promoter and RBS of this HK gene could result in population diversity and that the population would respond to the external signal in a mixed, rather than all-or-none fashion.

Our work has also general implications for the understanding of molecular interaction networks other than two-component systems. We have analysed a large-scale model of a complete sequence of events linking external signal sensing with gene expression and shown the emergence of population diversity that does not derive from multistability of the system, but rather from slow production of a constituent chemical species. This phenomenon is very likely to be present in molecular interaction networks in general. The case of TCS histidine kinase indicates that noise in the expression of a single gene producing an external signal sensor can result in population diversity and a mixed-mode population response to that external signal. Potential occurrence of this mechanism should be taken into account in studies of a wide range of signal transduction cascades both in bacterial and eukaryotic cells.

We show for the first time, to the best of our knowledge, the use of equation-free techniques to analyse a detailed model of a signal transduction and gene regulatory network. Our results demonstrate that this approach enables the application of the classical concepts of dynamical systems theory to the analysis of realistic stochastic models of molecular interaction networks of the cell. The calculation of the Jacobian is particularly useful as it provides insight into the stability of the behaviours observed in numerical realisations of stochastic dynamics. Understanding parameter dependencies in stochastic systems that are accessible only through direct numerical simulation is a major challenge. Hitherto, this has typically been attempted through time-consuming numerical experiments, without a systematic method for evaluating changes in the expected (probabilistic mean) system behaviour. Frequently, observation of a particular phenomenon in simulation trajectories brings little understanding of the underlying mechanism. Our work shows that equation-free methods provide a systematic and feasible solution to this problem. Our use of equation-free techniques to investigate stochastic phenomena in a biochemical reaction network of realistic scale demonstrates their potential for enabling greater insight into the behaviour of highly stochastic systems in biology, and also the challenges of scale that must be overcome in order to do so.

To summarise, our work provides insight into the mechanisms of emergence of phenotypic diversity in populations of genetically identical cells. Our successful use of equation-free methods in this context will motivate future applications of this approach for the analysis of the stochastic dynamics of molecular interaction networks.

## Methods

### Derivation of the evolution equations for the mean 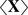


The rate of change with time of the vector of mean species numbers, 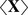, can be calculated from the chemical master equation
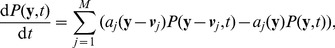
(71)where 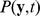 is the probability that 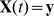 - see [24], for example - and 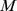 is the number of different types of chemical reaction in the system. The mean is given by 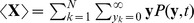, where 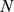 is the number of chemical species, and so it evolves according to
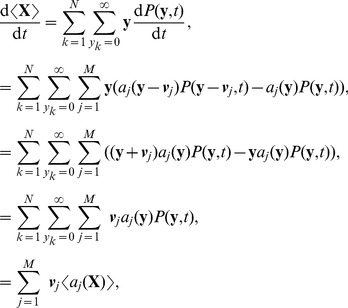
(72)where it should be noted that 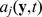 and 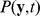 are defined to be zero if 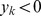 for any 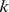.

### Properties of 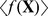 when 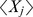 is zero

Note that if 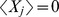 for some 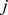, then we have
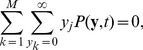
(73)and so since 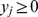, 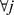 and 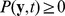, 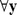, 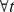, we must have 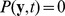 for all 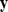 such that 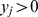. Then for any function 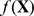 the mean is given by
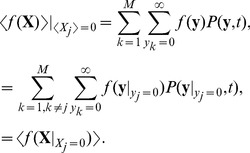
(74)


### Composition of the coarse time-stepper

The coarse time-stepper used in the equation-free method is composed from microscopic runs of the Gillespie algorithm in three stages:

1. **Lift:** A set of 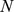 microscopic initial conditions 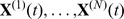 is obtained from the initial coarse variable 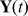. We note that while each 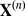 is a vector of natural numbers, the macroscopic variable 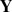 is a vector of reals. As a consequence, the lifting of a generic macroscopic component 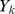 should return either 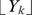 or 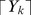 (where 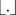 and 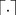 denote the floor and ceiling functions, respectively). In our implementation, the lifting of each component 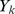 is achieved by drawing 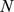 samples from the following Bernoulli distribution
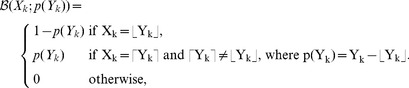
(75)As mentioned in the section on equation-free methods, the lifting operator introduces a closure approximation. The Dirac moment map used in [28] is a good choice when one is interested in the evolution equation of the first moment of the distribution, but it cannot be applied directly when the microscopic variables take discrete values. If the microscopic variables 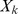 were real numbers, the lifting would be done using the Dirac measure 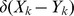. In our case, however, the microscopic variables 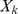 count the number of molecules of a given species, so we have to use a measure over the integers, and such measure is to be uniquely determined by its mean, hence our choice of the Bernoulli distribution with support 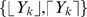. In order to make the problem tractable, we have also assumed that the distribution for 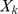 depends only on 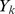, neglecting the effect of correlations between the numbers of particles of different species.
2. **Evolve:** The microscopic initial conditions are evolved forward with 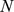 independent runs of the Gillespie algorithm, leading to the final conditions 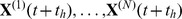. We use the modified tau-leaping Gillespie algorithm proposed by Cao et al. [38]. This modification of the tau-leaping scheme is adaptive in time and prevents the occurrence of negative populations in the reactants: in our computations, we deem a reaction critical if the number of permitted firings during the current time step is less than or equal to 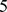 (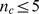). When the time step is too small, we run 100 iterations of the unmodified Gillespie algorithm before applying a tau-leap step. To reduce calculation time, we only calculate 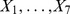 and 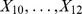, because the dynamics of 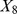 and 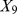 can be decoupled from the rest.
3. **Restrict:** The microscopic variables at time 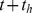 are averaged in order to obtain the final coarse variables 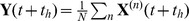. The restriction step is essentially an approximation of the definition (68).
